# Inflammation- and Gut-Homing Macrophages, Engineered to *De Novo* Overexpress Active Vitamin D, Promoted the Regenerative Function of Intestinal Stem Cells

**DOI:** 10.3390/ijms22179516

**Published:** 2021-09-01

**Authors:** Yi Xu, David J. Baylink, Huynh Cao, Jeffrey Xiao, Maisa I. Abdalla, Samiksha Wasnik, Xiaolei Tang

**Affiliations:** 1 Department of Medicine, Division of Regenerative Medicine, Loma Linda University, Loma Linda, CA 92354, USA; Dyxu@llu.edu (Y.X.); DBaylink@llu.edu (D.J.B.); swasnik@llu.edu (S.W.); 2 Division of Hematology and Oncology, Department of Medicine, Loma Linda University & Loma Linda University Cancer Center, Loma Linda, CA 92354, USA; hcao@llu.edu (H.C.); jeffreyxiao77@gmail.com (J.X.); 3 Division of Gastroenterology and Hepatology, University of Rochester Medical Center, Rochester, NY 14642, USA; Maisa_Abdalla@URMC.Rochester.edu; 4 Department of Veterinary Biomedical Sciences, College of Veterinary Medicine, Long Island University, Brookville, NY 11548, USA

**Keywords:** inflammatory bowel disease, vitamin D, CD11b^+^Gr1^+^ macrophages, Lgr5, intestinal stem cells

## Abstract

Inflammatory bowel disease (IBD) is a chronic inflammatory disease of the gut. Available drugs aim to suppress gut inflammation. These drugs have significantly delayed disease progression and improved patients’ quality of life. However, the disease continues to progress, underscoring the need to develop novel therapies. Aside from chronic gut inflammation, IBD patients also experience a leaky gut problem due to damage to the intestinal epithelial layer. In this regard, epithelial regeneration and repair are mediated by intestinal stem cells. However, no therapies are available to directly enhance the intestinal stem cells’ regenerative and repair function. Recently, it was shown that active vitamin D, i.e., 1,25-dihydroxyvitamin D or 1,25(OH)_2_D, was necessary to maintain Lgr5^+^ intestinal stem cells, actively cycling under physiological conditions. In this study, we used two strategies to investigate the role of 1,25(OH)_2_D in intestinal stem cells’ regenerative function. First, to avoid the side effects of systemic high 1,25(OH)_2_D conditions, we used our recently developed novel strategy to deliver locally high 1,25(OH)_2_D concentrations specifically to inflamed intestines. Second, because of the Lgr5^+^ intestinal stem cells’ active cycling status, we used a pulse-and-chase strategy via 5-bromo-2′-deoxyuridine (BrdU) labeling to trace the Lgr5^+^ stem cells through the whole epithelial regeneration process. Our data showed that locally high 1,25(OH)_2_D concentrations enhanced intestinal stem cell migration. Additionally, the migrated cells differentiated into mature epithelial cells. Our data, therefore, suggest that local delivery of high 1,25(OH)_2_D concentrations is a promising strategy to augment intestinal epithelial repair in IBD patients.

## 1. Introduction

Inflammatory bowel disease (IBD) is a chronic inflammatory disease of the gut. It is generally accepted that IBD is caused by aberrant immune responses to intestinal bacteria [1]. Ulcerative colitis (UC) and Crohn’s disease are the two main types of IBD and affect more than 3.6 million people in the United States and Europe [2,3]. IBD incidence continues to rise globally [4]. Current IBD management aims to suppress the underlying immunological mechanisms. Available drugs include immune-modulating drugs (e.g., 5-aminosalicylic acid), immunosuppressants (e.g., glucocorticoids, azathioprine, and 6-mercaptopurine), and most recently, biological medications (e.g., anti-tumor necrosis factor-α monoclonal antibody (infliximab)) [5,6]. These drugs have significantly delayed disease progression and improved patients’ quality of life. However, the above mediations only slow down the disease progression and are not a cure. Therefore, it is necessary to fully understand the mechanisms that may potentially lead to IBD cure.

In addition to chronic gut inflammation, IBD patients also experience a leaky gut problem due to the damage of the intestinal epithelial layer [7]. As mentioned above, a series of FDA-approved drugs are available to control chronic inflammation in IBD patients. However, no medications are explicitly aimed to repair the damaged intestinal epithelium. Therefore, leaky gut is still a challenge to IBD treatment. In this regard, epithelial regeneration and repair are mediated by intestinal stem cells [1,8]. However, intestinal stem cells’ regenerative mechanisms are far from being understood because of the delayed discovery of their specific biomarkers [9,10,11,12,13].

Concerning the intestinal stem cells, Peregrina K et al. recently showed that a lack of vitamin D signaling led to decreased Lgr5^+^ intestinal stem cell numbers in intestinal crypts [14]. Using in vitro organoid cultures, Fernandez-Barral A et al. demonstrated that human Lgr5^+^ intestinal stem cells expressed vitamin D receptor (VDR) [15]. In addition, 1,25(OH)_2_D, i.e., the active vitamin D, upregulated the expression of the genes associated with stemness in Lgr5^+^ intestinal stem cells [15]. Because Lgr5^+^ intestinal stem cells are the only actively cycling stem cells to regenerate intestinal epithelia under physiological conditions, these previous studies suggest that vitamin D regulates intestinal stem cells’ regenerative function.

For the role of 1,25(OH)_2_D in IBD treatment, several studies have demonstrated that systemic treatment with 1,25(OH)_2_D remarkably ameliorated IBD in mice [16,17,18]. However, clinical trials of systemic supplementation of various vitamin D forms have failed to generate anticipated therapeutic outcomes [19,20,21]. We hypothesized that the systemic treatment failure was due to the inability to provide the needed 1,25(OH)_2_D concentrations without inducing hypercalcemia (i.e., a higher blood calcium level than the physiological levels). Unfortunately, the hypercalcemia-inducing 1,25(OH)_2_D levels were essential for the effective suppression of gut inflammation. To test the above hypothesis, we proposed a novel strategy in which inflammation-homing CD11b^+^Gr1^+^ monocytes [22,23] and macrophages were transduced with a lentiviral vector to overexpress the cytochrome P450 family 27 subfamily B member 1 (CYP27B1) [24]. Such engineered cells were named MAC-CYP cells. The CYP27B1 gene encodes the 25-hydroxyvitamin D 1α-hydroxylase (1α-hydroxylase) that physiologically converts 25-hydroxyvitamin D (i.e., the primary vitamin D form in the blood) into the active 1,25(OH)_2_D. We reasoned that the MAC-CYP cells would home specifically to inflamed intestines and *de novo* produce locally high 1,25(OH)_2_D concentrations, suppressing the intestinal inflammation in IBD patients. Because the high 1,25(OH)_2_D concentration was local, it should not cause hypercalcemia. Indeed, we showed that the MAC-CYP cells, when administered intravenously, effectively ameliorated experimental colitis [24]. Additionally, the colitis amelioration was associated with the suppression of proinflammatory cytokines (e.g., TNF-α, IL-1β, IL-6, IL-12, IL-23, IFN-γ, and IL-17) and the enhanced expression of intestinal junction proteins (e.g., claudin-1 and zonula occludens) in inflamed colons [24]. Notably, the MAC-CYP cells did not induce hypercalcemia [24], supporting our original hypothesis.

This study hypothesized that locally high 1,25(OH)_2_D concentrations would enhance intestinal stem cells’ regenerative functions. To test this hypothesis, we used two strategies. First, we used the MAC-CYP cells to deliver locally high 1,25(OH)_2_D concentrations specifically to inflamed intestines. Second, because the Lgr5^+^ intestinal stem cells are actively proliferating, we used a pulse-and-chase strategy via 5-bromo-2′-deoxyuridine (BrdU) labeling to trace Lgr5^+^ stem cells through the whole epithelial regeneration process.

## 2. Results

### 2.1. Generation of Inflammation- and Gut-Homing MAC-CYP Cells In Vitro

**Figure 1:**
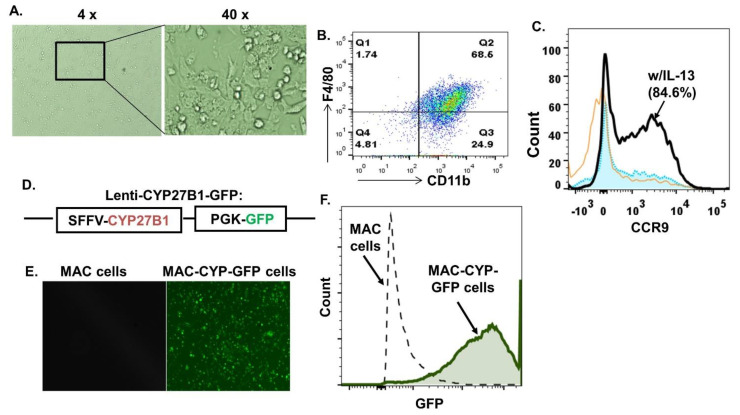
Generation of primary CD11b^+^Gr1^+^CCR9^+^ macrophages (MAC cells) engineered to overexpress CYB27B1 (MAC-CYP cells) in vitro. The MAC cells were generated as described in Materials and Methods. (**A**) 4× (left panel) and 40× (right panel) phase bright images of primary MAC cells are shown. (**B**) A FACS plot shows macrophage markers’ expression, i.e., CD11b and F4/80. (**C**) A FACS plot shows the expression of CCR9 in the presence or absence of IL-13. Orange line plot: without IL-13, black line plot: with IL-13 (w/IL-13), filled blue plot: isotype control. (**D**) Schematic diagram of the lentiviral plasmid of lenti-CYP27B1-GFP. SSFV and PGK are promoters. (**E**) Representative images of MAC (left panel) and MAC-CYP-GFP cells (right panel) under the fluorescence field. (**F**) A FACS plot shows GFP expression in the MAC and MAC-CYP cells.

A recent study showed that a combination of interleukin-13 (IL-13), granulocyte-colony-stimulating factor (G-CSF), and granulocyte-macrophage-colony-stimulating factor (GM-CSF) induced CD11b^+^Gr1^+^ cells that migrated to allo-priming sites and robustly suppressed graft-versus-host disease when administered in animals [25]. The above findings were consistent with a previous report that Gr1^+^ monocytes homed specifically to inflamed sites [22]. Interestingly, the CD11b^+^Gr1^+^ cells induced by the above protocol also expressed a high level of CCR9 [25]. CCR9 is not only a gut-homing receptor; it is also essential for inflammatory cell recruitment, as demonstrated in several inflammatory disease models [26,27,28,29,30,31,32]. Based on the above previous findings, we modified the MAC cells we used previously [24]. Namely, we isolated CD11b^+^Gr1^+^ monocytes from mouse bone marrow using magnetic beads and cultured them in the presence of the above three cytokines for seven days to generate the MAC cells. Fluorescence-activated cell sorting (FACS) analysis confirmed the expression of macrophage markers (i.e., CD11b and F4/80) (Figure 1A,B) and CCR9 (Figure 1C) in the MAC cells. Interestingly, we found that IL-13 was necessary for CCR9 upregulation (Figure 1C).

Next, we performed the lentiviral transduction to overexpress the CYP27B1 ectopic gene in the MAC cells. CYP27B1 expression was assessed by GFP expressed by the same lentiviral construct (Figure 1D). The FACS data demonstrated that over 90% of the MAC cells were successfully transduced (MAC-CYP-GFP cells) (Figure 1E,F).

### 2.2. Intraperitoneally Administered MAC-CYP Cells Migrated Specifically into Inflamed Colons

Although the MAC-CYP cells potentially have two properties, i.e., inflammation-homing (via CCR9 and CCR2, among others) and gut-homing (via CCR9) [22,25], intravenous delivery may still take time for the cells to reach the inflamed gut. Hence, we used intraperitoneal delivery of the MAC-CYP cells rather than previously described intravenous delivery [24]. Because of being in an enclosed environment with proximity to the intestinal tissues, intraperitoneally administered MAC-CYP cells were assumed to stay concentrated near intestines for a more extended period and hence homed efficiently into inflamed intestinal tissues.

**Figure 2:**
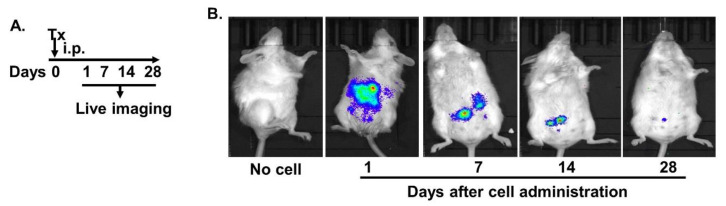
Intraperitoneally administered MAC-CYP cells could be detected in the peritoneal cavity for at least 28 days. (**A**) Experimental design. Tx: MAC-CYP-Luciferase cells. (**B**) Representative live images show luciferase activity on days 1, 7, 14, and 28 following the cell administration.

Accordingly, we first examined the length of time that the intraperitoneally administered MAC-CYP cells could stay concentrated in the peritoneal cavity. To answer this question, we generated the MAC-CYP cells with a lentiviral vector that expressed both 1α-hydroxylase and luciferase (MAC-CYP-Luciferase cells). Subsequently, 4 × 10^6^ MAC-CYP-Luciferase cells per mouse were administered into the peritoneal cavity of healthy Balb/c mice (Figure 2A). On days 1, 7, 14, 28 following the cell administration, the mice were monitored for the presence of luciferase by live imaging. Our data showed that luciferase activity could be detected in the peritoneal cavity for at least 28 days (Figure 2B).

**Figure 3:**
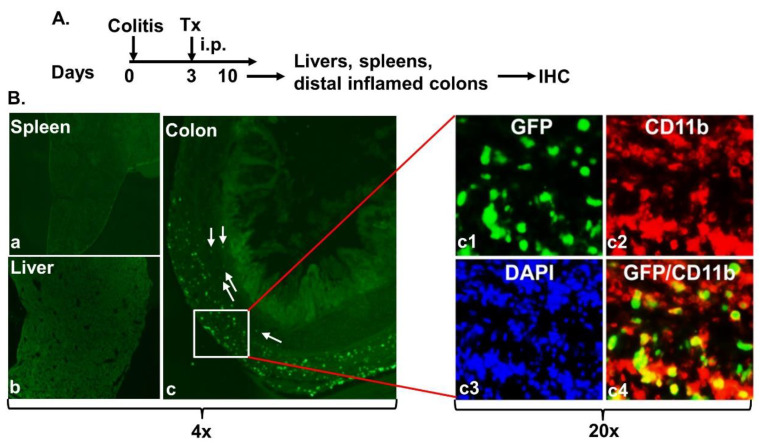
Intraperitoneally administered MAC-CYP cells migrated specifically into inflamed colons. (**A**) Experimental design. Tx: MAC-CYP-GFP cells. (**B**) Representative 4× images of MAC-CYP-GFP-treated animals show immunostaining of GFP and CD11b of the spleen (**a**); liver (**b**); and distal inflamed colons (**c**). Arrows indicate GFP^+^ cells that have migrated to the proximity of crypts. The boxed area is further enlarged (20×) for viewing the staining of GFP (**c1**), CD11b (**c2**), and DAPI (**c3**). (**c4**) shows GFP and CD11b merged staining.

Next, we determined the migration of intraperitoneally administered MAC-CYP cells. Thus, Balb/c mice were induced for experimental colitis as described in Materials and Methods (Figure 3A). Three days after the colitis induction, 4 × 10^6^ MAC-CYP-GFP cells per mouse were administered into the peritoneal cavity (Tx). Seven days after the cell administration, frozen sections of spleens, livers, and distal inflamed colons were analyzed for GFP and CD11b expression by immunohistochemistry (IHC). Our data showed that the GFP^+^ cells were present deep in the inflamed colon tissues but not spleens and livers [Figure 3B(a–c)]. Additionally, some GFP^+^ cells (arrows) had migrated to the proximity of crypts [Figure 3B(c)]. Moreover, most migrated GFP^+^ cells were CD11b^+^, a marker for macrophages [Figure 3B(c1–c4)].

The intraperitoneally-administered MAC-CYP cells’ specific migration into inflamed colons was more impressive than what we observed in our previous study [24]. Next, we wanted to determine whether the intraperitoneally administered MAC-CYP cells also effectively suppressed experimental colitis. We previously evaluated the effects of the MAC-CYP cells on colitis induced by dextran sulfate sodium [24]. This study assessed the impact of the MAC-CYP cells on colitis induced by 2,4,6-trinitrobenzenesulfonic acid (TNBS). We chose TNBS colitis because it is a well-established Th1-mediated disease, as described previously [33]. In addition, using the adoptive transfer of Th17 cells deficient in interferon-γ (IFN-γ) (IFN-γ is a Th1 cytokine), a recent study demonstrated that Th1 cells were essential for mediating colitis [34].

**Figure 4:**
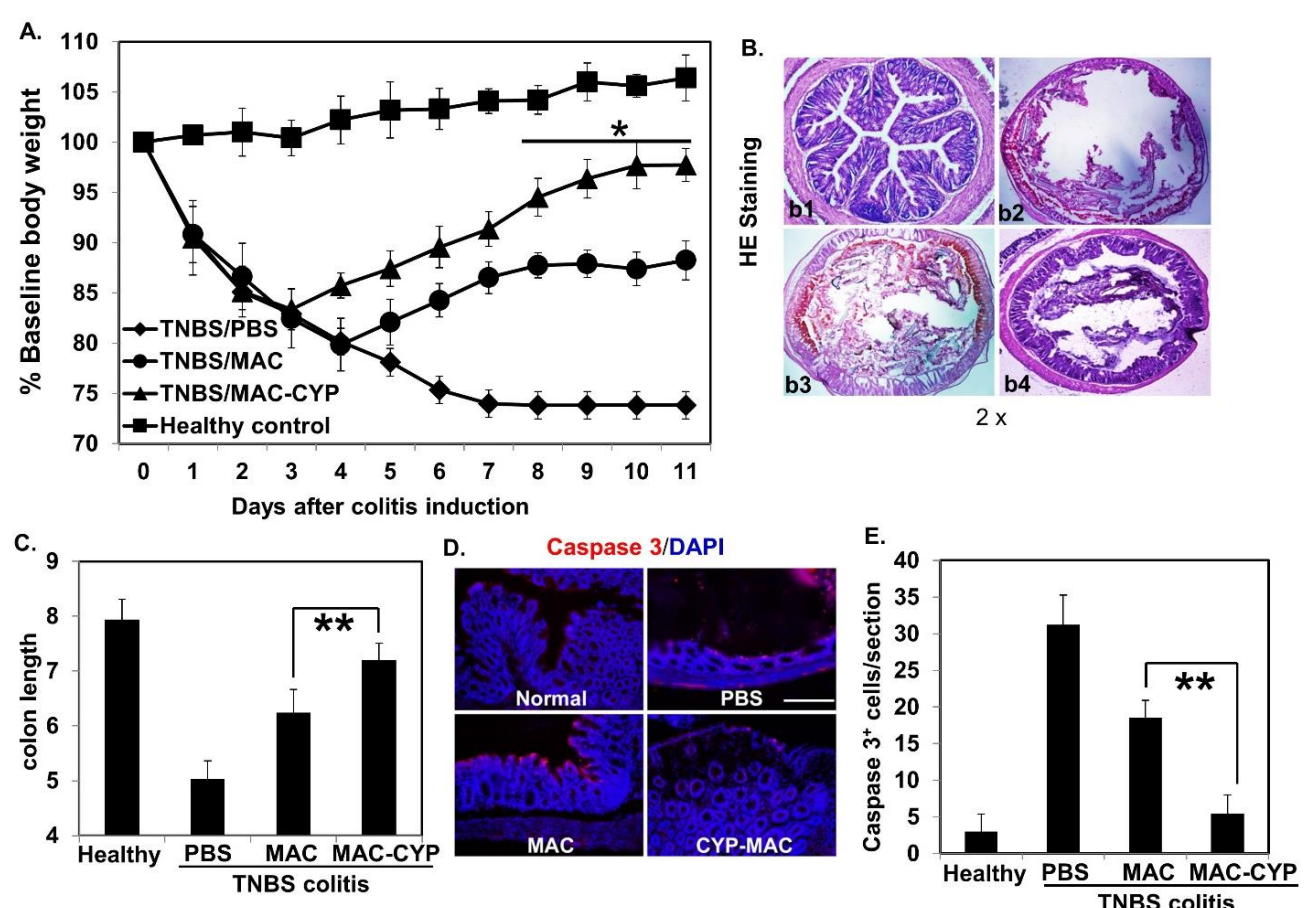
Intraperitoneally administered MAC-CYP cells ameliorated TNBS colitis. TNBS colitis was induced as described in Section 4—Materials and Methods. (**A**) % Baseline body weights are shown. (**B**) Representative images of HE staining. (**b1**): normal; (**b2**): TNBS/PBS; (**b3**): TNBS/MAC; (**b4**): TNBS/MAC-CYP. (**C**) Quantitative analysis of colon length. (**D**) Immunochemistry staining of caspase 3 and DAPI. Scale bar = 10 μm. (**E**) Cumulative data of caspase 3^+^ cells per tissue section from (**D**). Where applicable, data are means ± SEM (*n* = 5) and are representative of two independent experiments. * *p* < 0.05; ** *p* < 0.01.

Accordingly, TNBS colitis was induced in Balb/c mice. Three days later, the mice intraperitoneally received one of the following treatments: PBS, the MAC cells, and the MAC-CYP cells. Additionally, healthy animals were also included as a control. Similar to previous findings [24], animals without treatments experienced bodyweight loss. The MAC cells themselves reduced the bodyweight loss. However, the MAC-CYP cells had a more significant effect than the MAC cells (Figure 4A). It is essential to mention that the MAC cells’ therapeutic effects are expected because they are myeloid suppressors [25].

On day 10 (7 days post the treatments), some of the mice were sacrificed. The colon samples were collected and processed for histological analyses. Consistent with the bodyweight data, this analysis showed that the colon epithelia in the PBS-treated mice were severely damaged. Those in the MAC-treated mice displayed less damage, and those in the MAC-CYP-treated mice remained relatively intact (Figure 4B). Moreover, colon length analysis revealed similar therapeutic effects (Figure 4C). Finally, staining of caspase 3 (an apoptosis marker) demonstrated that both PBS- and MAC-treated colons showed significant staining. In contrast, MAC-CYP treatment resulted in staining showing close to normal healthy intestines (Figure 4D,E).

### 2.3. Intraperitoneally Administered MAC-CYP Cells Accelerated Intestinal Stem Cell Migration

**Figure 5:**
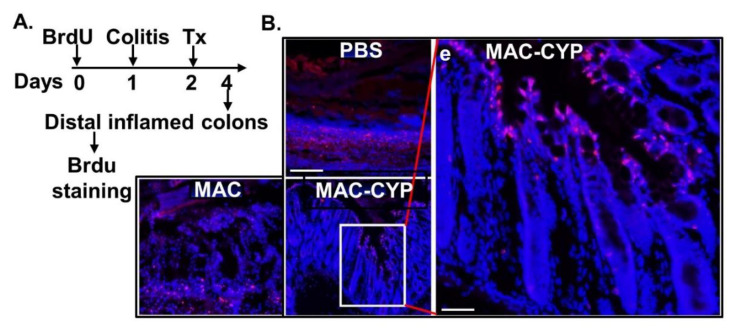
Intraperitoneally administered MAC-CYP-GFP cells accelerated intestinal stem cell migration in inflamed colons. (**A**) Experimental design. (**B**) Representative images show distal inflamed colons from the animals induced for TNBS colitis and treated with PBS, MAC, or MAC-CYP cells (scale bar = 10 μm) for two days (**e**) is an enlarged view of the boxed area, scale bar = 40 μm).

Having determined the specific migration and colitis suppression properties, we examined the impact of the MAC-CYP cells on the intestinal stem cells. In this regard, intestinal stem cells regenerate and repair the intestinal wall through gradual migration and differentiation from intestinal crypts to the epithelial villi’s tip [13]. To study intestinal stem cell migration, we took advantage of the proliferating nature of the Lgr5^+^ intestinal stem cells under physiological conditions [35]. Namely, we first intraperitoneally administered BrdU every 2 hours for 12 hours to pre-label these proliferating cells and then chased the BrdU^+^ cells’ migration afterward (Figure 5A). It is worth mentioning that BrdU has a half-life of approximately 40–60 mins in vivo [36]. Therefore, proliferating cells resulting from inflammation after the labeling process should not be exposed to BrdU, eliminating the possibility of off-target labeling. Thus, on the next day following the BrdU labeling, the mice were induced for TNBS colitis. One day later, the mice intraperitoneally received one of the following treatments: PBS, 4 × 10^6^ MAC cells, and 4 × 10^6^ MAC-CYP cells. Two days following the cell treatment, distal inflamed colons were analyzed for BrdU^+^ cells by IHC. Our data showed that colitis caused severe damage in the intestinal epithelium (Figure 5B, PBS). Furthermore, the MAC cells provided some degree of protection from the damage (Figure 5B, MAC), consistent with the immune suppressive function of CD11b^+^Gr1^+^ cells [25]. Furthermore, the MAC-CYP cells provided much more robust protection from damage (Figure 5B, MAC-CYP). Notably, four days after the BrdU labeling and two days after the cell treatment, most of the BrdU^+^ cells had already migrated to the top of the epithelium [Figure 5B(e)].

**Figure 6:**
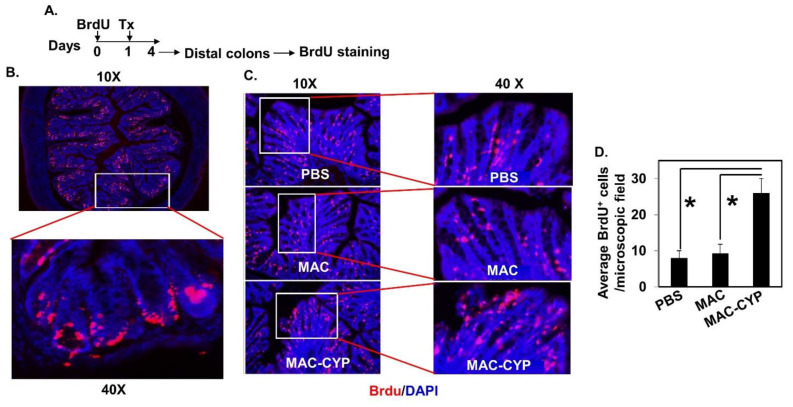
Intraperitoneally administered MAC-CYP cells accelerated intestinal stem cell migration in normal healthy animals. (**A**) Experimental design. (**B**) Representative images show BrdU staining of non-treated distal colon tissues immediately after BrdU labeling on day 0. Upper panel: 10×; lower panel: 40×. (**C**) Representative images of distal colon tissues four days after BrdU labeling and three days after the animals were treated with PBS; MAC; or MAC-CYP (day 4). Left panel: 10× images; right panel: 40× images. (**D**) Average numbers of BrdU^+^ cells of three randomly chosen microscopic fields at the top 1/3 areas of the colon epithelia from PBS-, MAC-, and MAC-CYP-treated animals are shown. * *p* < 0.05. ANOVA test, *n* = 5.

In the above TNBS colitis model, the severe damage of intestinal epithelia caused unclear staining of intestinal stem cells in the MAC cell-treated animals. Consequently, it was difficult to conclude whether the MAC-CYP cell treatment accelerated intestinal stem cell migration. Hence, we performed a similar experiment in normal healthy animals. Accordingly, normal healthy Balb/c mice were pre-labeled with BrdU and received treatments (Figure 6A). Three days after the cell treatment, frozen sections of distal colons were stained for BrdU^+^ cells by IHC. Our data showed that the intestinal stem cells were successfully pre-labeled with BrdU immediately following the labeling procedure (Figure 6B). Three days later, most pre-labeled BrdU^+^ cells left the crypts and started migration (Figure 6C, PBS). In addition, treatment with the MAC cells did not significantly affect this migration (Figure 6C, MAC, and Figure 6D). In contrast, the animals treated with the MAC-CYP cells showed substantially more BrdU^+^ cells in the top one-third of the intestinal epithelia (Figure 6C, MAC-CYP, and Figure 6D).

### 2.4. Migrated Intestinal Stem Cells Differentiated into Mature Epithelial Cells

**Figure 7:**
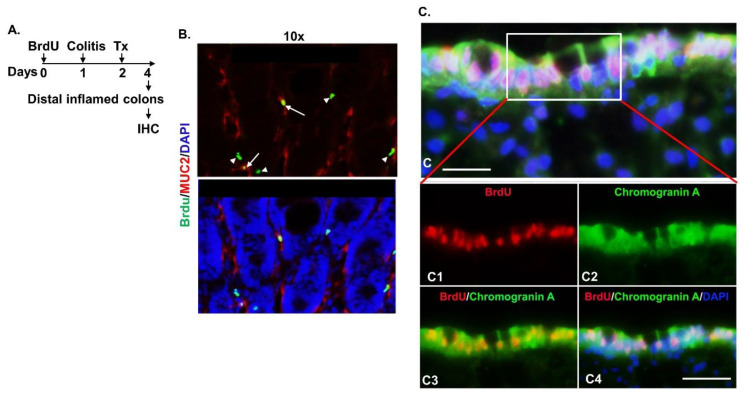
Migrated intestinal stem cells differentiated into mature epithelial cells. (**A**) Experimental design. (**B**) Representative images show distal inflamed colon tissues that were stained for MUC2 (i.e., a marker for goblet cells). Arrows: co-stained cells. Arrowheads: single-stained cells. (**C**) Representative images show distal inflamed colon tissues that were stained for chromogramin A (i.e., a marker for enteroendocrine cells). (**c**): scale bar = 20 μm. (**c1**–**c4**): scale bar = 40 μm.

Usually, migrating intestinal stem cells differentiate into mature intestinal epithelial cells to regenerate or repair the intestinal wall [13]. To determine whether the migrated BrdU^+^ intestinal stem cells differentiated into mature epithelial cells, we used IHC to analyze the distal inflamed colon tissues from the Balb/c mice treated with the MAC-CYP cells as described in Figure 5 (Figure 7A). Our data showed that the migrated BrdU^+^ cells were co-stained with MUC2 (i.e., a marker for goblet cells) (Figure 7B) and chromogranin A (i.e., a marker for enteroendocrine cells) (Figure 7C). Therefore, our data indicated that the migrated BrdU^+^ stem cells differentiated into mature epithelial cells.

## 3. Discussion

The current approaches to IBD treatment consist of anti-inflammatory and immunosuppressant drugs. However, IBD is characterized not only by the sub-mucosal accumulation of inflammatory cells but also by the severe damage of the epithelial layer [7]. Thus, “mucosal healing” is the most significant prognostic factor for long-term remission in IBD patients. The accomplishment of epithelial regeneration is critically required to improve the treatment for IBD. This study provides evidence that 1,25(OH)_2_D, *de novo* synthesized by the MAC-CYP cells, enhances intestinal stem cells’ regenerative function. Hence, we, for the first time, describe one more potential beneficial role of 1,25(OH)_2_D in IBD patients in addition to the previously described inflammation suppression, stimulation of antimicrobial peptides, and tight junction protection [24,37].

We previously showed that 1,25(OH)_2_D suppressed inflammatory cell function and maintained epithelium tight junctions in the inflamed gut [24]. Because inflammation and epithelial tight junction damage can impact intestinal stem cells [38,39], the previously observed 1,25(OH)_2_D effects on intestinal stem cells might have been indirect due to inflammation suppression and tight junction protection. This current study showed that the MAC-CYP cells also had effects in healthy animals (Figure 6), similar to the colitic animals (Figure 5). Because healthy animals were devoid of any intestinal abnormalities, our data suggested that the 1,25(OH)_2_D-mediated actions directly affected intestinal stem cells.

Our data are consistent with previous reports that VDR plays a critical role in maintaining stem cells’ normal physiological function in different organs [14,40,41]. Interestingly, whereas 1,25(OH)_2_D maintains normal stem cells’ stemness, it inhibits stem cell growth in malignant tissues [15,42,43,44]. Our study showed that 1,25(OH)_2_D, *de novo* synthesized by the MAC-CYP cells, enhanced intestinal stem cells’ migration and differentiation under physiological and inflammatory conditions.

It is important to mention that the intestinal stem cells detected in our experiments should be mostly Lgr5^+^ cells. Supporting evidence for this preceding claim is that only the Lgr5^+^ cells actively proliferate under physiological conditions [13] and are therefore labeled by BrdU (Figure 6B). In addition, in our labeling protocol, BrdU was injected 24 hours before any interventions. Because of a short half-life (40–60 min) in mice [45], proliferating cells after colitis induction and cell administration should not be exposed to BrdU. Therefore, BrdU^+^ cells, after labeling, represent only the proliferating cells under the physiological conditions, i.e., mostly Lgr5^+^ intestinal stem cells (Figure 6B). However, future studies using mice in which Lgr5^+^ cells are labeled specifically with fluorescence are needed to solidify this conclusion.

**Figure 8:**
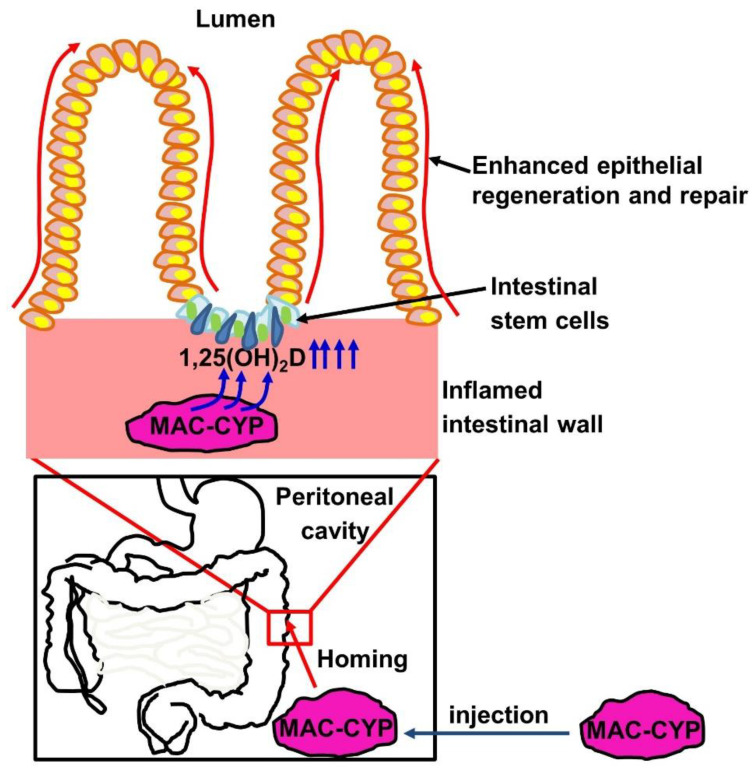
A model for the role of locally high 1,25(OH)_2_D concentrations in intestinal stem cells’ regenerative function. Inflammation- and gut-homing macrophages were engineered to overexpress the 1α-hydroxylase (MAC-CYP cells). When injected into inflammatory bowel disease patients’ peritoneal cavity, the MAC-CYP cells migrate specifically into inflamed intestines. Subsequently, the MAC-CYP cells *de novo* produce locally high 1,25(OH)_2_D concentrations to enhance intestinal stem cells’ migration and differentiation, thereby augmenting intestinal epithelial repair.

Based on our findings, we propose a model for the role of locally high 1,25(OH)_2_D concentrations in intestinal stem cells’ regenerative function (Figure 8). In this model, inflammation- and gut-homing macrophages are engineered to overexpress the 1α-hydroxylase. When injected into the peritoneal cavity of inflammatory bowel disease patients, the engineered macrophages migrate specifically into inflamed intestines. Subsequently, the engineered macrophages *de novo* produce locally high 1,25(OH)_2_D concentrations to enhance intestinal stem cells’ migration and differentiation, thereby augmenting intestinal epithelial repair.

A limitation of this study was that the role of 1,25(OH)_2_D in intestinal stem cells was deduced by comparing the MAC-CYP cells with the MAC cells. Although the MAC-CYP cells, but not the MAC cells, could *de novo* synthesize locally high 1,25(OH)_2_D concentrations in inflamed gut, direct evidence using conditional VDR knockout in intestinal stem cells will definitively confirm this novel function of 1,25(OH)_2_D.

In conclusion, we have demonstrated that inflammation- and gut-homing CD11b^+^Gr1^+^ macrophages, engineered to *de novo* synthesize high 1,25(OH)_2_D concentrations, specifically migrate into inflamed intestines to promote the regenerative function of intestinal stem cells. This study is an extension of previous findings that such engineered macrophages effectively suppress the inflammatory response (e.g., TNF-α, IL-β, IL-6, IL-12, IL-23, IFN-γ, IL-17) and enhance the intestinal epithelial barrier function [24]. The novel function described in this study further underscores that 1,25(OH)_2_D is a promising therapy for IBD. Such therapy can be achieved through local delivery without the risk of hypercalcemia.

## 4. Materials and Methods

### 4.1. Mice

BALB/c mice were purchased from The Jackson Laboratory (Bar Harbor, ME) and housed in a specific pathogen-free animal facility at Loma Linda University (LLU). All mice used were 6–8 weeks old and allowed to acclimate for a minimum of 5 days before any experimentation. All in vivo animal study protocols were reviewed and approved by LLU Institutional Animal Care and Use Committee and the Animal Care and Use Review Office of the US Army Medical Research and Materiel Command of the Department of Defense.

### 4.2. Induction of Experimental Colitis

Experimental colitis was induced by intracolonic injection of 2,4,6-trinitrobenzenesulfonic acid (TNBS) (Sigma-Aldrich) according to a previously reported method [33,46]. Briefly, a 2.5% TNBS working solution in 50% ethanol, was prepared by dissolving 5% TNBS (Sigma-Aldrich, St. Louis, MO, USA) in an equal volume of absolute ethanol. Subsequently, BALB/c mice were anesthetized by intraperitoneal (*IP*) injection of 100 μL ketamine (12 mg/mL)/xylazine (1.6 mg/mL) solution per 10 g body weight. A 3.5 French gauge catheter connected to a 1 mL syringe was then carefully inserted into colons such that the catheter tip was 4 cm proximal to the anus. Then, 150 μL of the TNBS working solution was administered. Mice were then kept in a vertical position for 60 s and returned to their cages.

### 4.3. Preparation of Lentiviruses

Lentiviruses were prepared as previously described [37]. For the generation of a lentivirus that carried one of the lentiviral transfer plasmids, 293T cells were cultured in complete Dulbecco’s modified Eagle’s medium (DMEM) containing 10% FBS, 100 U/mL penicillin/streptomycin, 0.05 mM 2-mercaptoethanol (ME), 1 mM sodium pyruvate, 0.1 mM nonessential amino acid, and 2 mM L-glutamine. When the cells reached 70–80% confluence, culture media were replenished, and a transfection solution containing an envelope, packaging, and the transfer plasmid was added to the cells dropwise. The cells were then cultured at 37 °C and 5% CO_2_ for 24 h, and the transfection solution was replaced with a different DMEM culture that contained 4% FBS, 100 U/mL penicillin/streptomycin, and 20 mM HEPES. After the cells were cultured at 37 °C and 5% CO_2_ for 48 h, supernatants were collected, filtered through a 0.45 μm filter, and centrifuged at 4800× *g* at 4 °C for 24 h. The virus pellet was reconstituted in PBS containing 5% glycerol and titrated by a GFP-based FACS method. The typical titer of a virus was 10^8^–10^9^ transducing units/mL.

### 4.4. Generation of CD11b^+^Gr1^+^CCR9^+^ Macrophages (MAC Cells) from Bone Marrow and Lentivirus Transduction

The MAC-CYP cell generation was slightly modified from a previous report [25]. Briefly, after lysis of the red blood cells, bone marrow nucleated cells were collected and used for purification of CD11b^+^Gr1^+^ monocytes using the MACS technique. Gr1^+^ cells were positively selected using APC-conjugated anti-Gr1 antibody and anti-APC Microbeads (Miltenyi Biotec, Bergisch Gladbach, Germany). The positively selected cells were cultured in RPMI medium 1640 (Invitrogen) containing 10% FBS, human GM-CSF (R&D Systems), human G-CSF (R&D Systems), and human IL-13 (R&D Systems), each at 100 ng/mL for seven days. After seven days’ culture, the cells were transduced with indicated lentiviral vectors at a multiplicity of infection (MOI) of 5. Twenty-four hours later, the virus was removed, and culture media were replenished. The cells were cultured for another 24 h and examined for transduction efficiency under a fluorescence microscope. When necessary, the above transduction procedure was repeated one more time before use.

### 4.5. Intraperitoneal Administration of the MAC-CYP Cells

The MAC-CYP cells (4 × 10^6^ cells in 100 μL PBS) were intraperitoneally injected into mice as indicated in each experimental design. No substrates (i.e., 25(OH) D) were fed to the transferred mice. Engraftment was assessed by live imaging or immunohistochemistry (IHC).

### 4.6. Fluorescence-Activated Cell Sorting (FACS) Analysis

FACS analysis was performed as previously described (22, 23). Briefly, ∼0.5–1 × 10^6^ cells in 100 μL FACS buffer (PBS containing 1% FBS and 0.05% sodium azide, Sigma, St. Louis, MO, USA) were stained with various fluorescence-conjugated antibodies specific for the cell surface proteins of interest at 4 °C for 30 min. The surface-stained cells were then fixed and permeabilized using BD Pharmingen Cytofix/Cytoperm buffer. The cells were then stained with different fluorescence-conjugated antibodies specific for the intracellular proteins of interest at 4 °C for 30 min in BD Perm/Wash buffer. Finally, the cells were washed twice in the Perm/Wash buffer and twice in the FACS buffer before being analyzed on a BD FACSAria II. Data were analyzed using FlowJo software (Flowjo, Ashland, OR, USA).

### 4.7. Live Imaging of MAC-CYP-Luciferase Cells In Vivo

MAC-CYP-Luciferase cells (4 × 10^6^) were intra-peritoneally (I.P.) injected into mice. On days 1, 7, 14, and 28, the mice were anesthetized with 2% pentobarbital sodium (50 mg/kg), and I.P. injected with D-luciferin (150 mg/kg body weight) 10 min before imaging. Then, the animals were placed in the imaging chamber with a high-resolution CCD camera (Perkin Elmer IVIS Lumina III, Waltham, MA, USA) to visualize the MAC-CYP-Luciferase cells.

### 4.8. Histological Analysis

After being flushed with cold PBS, spleens, livers, or colons were fixed in 4% paraformaldehyde. The fixed tissues were then embedded within the optimal cutting temperature compound and mounted on a chuck in a cryostat for sectioning. The tissues were cut into 10 μm thick frozen sections, which were collected by adhering to slides and stored at −20 °C. BrdU administration and detection were performed as previously described [47]. Briefly, BrdU labeling (50 mg/kg) (Cat#: B23151, Invitrogen, Waltham, MA, USA) was performed according to our experimental designs. Tissue sections were analyzed using IHC. The following antibodies were used: anti-BrdU (Cat#: ab8152, Abcam, Cambridge, UK), anti-chromogranin A (Cat#: ab15160, Abcam, Cambridge, UK), anti-GFP (Cat#: ab6673, Abcam, Cambridge, UK), anti-CD11b (Cat#: ab133357, Abcam, Cambridge, UK), and anti-caspase 3 (Cat#: CST 9662S, Danvers, MA, USA).

### 4.9. Statistical Analysis

Statistical analyses were performed using GraphPad Prism software (GraphPad Software, San Diego, CA, USA). For experiments that contained more than two groups, ANOVA tests were used. For experiments that contained only two groups, Student’s *t*-tests were used. Data were presented as means ± SEM. Results were considered significant when the *p*-value was < 0.05.

## Data Availability

The datasets used and analyzed in the current study are available from the corresponding author.
